# Snapshot of antimicrobial stewardship programs in the hospitals of Pakistan: findings and implications

**DOI:** 10.1016/j.heliyon.2019.e02159

**Published:** 2019-07-25

**Authors:** Zikria Saleem, Mohamed Azmi Hassali, Furqan Khurshid Hashmi, Brian Godman, Zakkiudin Ahmed

**Affiliations:** a School of Pharmaceutical Sciences, Universiti Sains Malaysia, Malaysia; b Rashid Latif College of Pharmacy, Lahore, Pakistan; c University College of Pharmacy, University of the Punjab, Lahore, Pakistan; d Department of Clinical Pharmacology, Karolinska Institute, Stockholm, Sweden; e Strathclyde Institute of Pharmacy and Biomedical Sciences, Strathclyde University, Glasgow, UK; f Health Economics Centre, University of Liverpool Management School, Liverpool, UK; g Ripha Institute of Healthcare Improvement & Safety, Ripha University, Pakistan

**Keywords:** Epidemiology, Infectious disease, Microbiology, Public health, Antimicrobial stewardship, Antimicrobial resistance, Pakistan

## Abstract

**Objective:**

We are unaware of the extent of antimicrobial stewardship programs (ASPs) among hospitals in Pakistan, which is a concern given the population size, high use of antibiotics across sectors and increasing antimicrobial resistance (AMR) rates. Consequently, we sought to address this by undertaking a comprehensive survey.

**Method:**

In this cross-sectional observational study in Punjab, an instrument of the measure was developed based on health care facility characteristics and ASPs after an extensive literature review**.** The questionnaire was circulated by mail or through drop off surveys to medical superintendents or directors/heads of pharmacy departments of hospitals.

**Results:**

Out of 254, a total of 137 hospitals fully completed the questionnaire - 11 primary, 65 secondary, 46 tertiary and 15 specialized hospitals. The use of antimicrobial prescribing guidelines (68.7%), provision of infectious diseases consultation services (66.4%), clinical pharmacy service (65.7%), use of drug and therapeutics committees to approve antimicrobial prescribing (65.5%), regular audit by doctors on antimicrobial prescribing (54.1%) and use of a restricted formulary for antimicrobial (50.4%) were the most common ASPs. However, most of these activities were only somewhat or moderately successful. Whereas, electronic antimicrobial prescribing approval systems (15.3%), using a sticker to notify prescribers regarding the need to obtain approval for the antimicrobial prescribed (16.1%) and participation in the national antimicrobial utilization surveillance program (19.7%) were only seen in a few hospitals.

**Conclusion:**

Study inferred that there are inadequate ASPs in the hospitals of Pakistan. A multidisciplinary approach, clinical leadership and availability of motivated and trained individuals are essential elements for the success of future ASPs.

## 1 Introduction

In this unending battle of microbes against antimicrobials, the microbes appear to be winning, and the pipeline of new antimicrobials is near to the end. Antimicrobial resistance (AMR) due to the inappropriate use of antibiotics is a rising threat, which has caught the attention of international and national organizations [1, 2, 3]. Reducing antimicrobial resistance while maintaining the efficacy of antimicrobials is the dream and the instigation of antimicrobial stewardship programs (ASP) appears the best way to achieve this goal. The United Nations General Assembly (UNGA) convention held on 21 September 2016 discussed progress towards Sustainable Development Goals (SDGs) 2030 including concerns with the shortage of antimicrobials as a consequence of AMR [4]. The World Health Organization (WHO) surveillance of resistance to antimicrobials also showed growing AMR along with declining susceptibility of current antimicrobials [5].

Current estimates suggest that approximately 25%–45% of in-patients in hospitals are prescribed antimicrobials whilst in hospitals, higher though in some countries especially those with high rates of HIV, and around 30–50% of antimicrobial use is seen as irrational [6, 7, 8]. Reducing AMR while maintaining the efficacy of antimicrobials is a key goal among countries, especially lower and middle-income countries (LMICs) with high rates of infectious diseases and AMR [9]. ASPs are one way to improve antibiotic utilization in hospitals [9, 10]. The Infectious Disease Society of America (IDSA) and the Society of Healthcare Epidemiology of America (SHEA) endorse ASPs to develop and quantify the rational use of antibacterials through assessing current use against recommended guidelines [11, 12]. The Center for Disease Control and Prevention (CDC) and SHEA both promote the rational use of antimicrobials [13]. In line with IDSA, SHEA and CDC, The Joint Commission (TJC) also recommended features to reduce AMR and irrational antimicrobial use [14]. The World Health Assembly also recommended ASPs for all health-care facilities [5]. ASPs showed effectiveness to subside the occurrence of microbial infections and colonization with carbapenem-resistant and extended-spectrum β-lactamase-producing Gram-negative bacteria, methicillin-resistant *Staphylococcus aureus*, multidrug-resistant Gram-negative bacteria and *C difficile* [15]. A recent systematic review also demonstrated the positive effect of ASPs with improving antibiotic use in hospitals [16]. ASPs with multidisciplinary groups are hardly seen outside of health care settings of high income countries (HIC) [15]. This is exemplified by the lack of AMS programmes among hospitals in LMICs [17]. This may be due to the difficulties and challenges with implementing ASPs in LMICs including manpower and resource issues [9]. At this moment, the international community needs to step up when it comes to financing national action plans that co-prioritize infection prevention, antimicrobial resistance (AMR) and ASP [18]. Irrational antibiotic prescribing and the continuous development of resistant infections are both serious issues faced by Pakistan [2]. We are unaware of the extent of ASP among hospitals in Pakistan, which is a concern given the population size, high use of antibiotics across sectors and increasing AMR rates. Consequently, we sought to address this by undertaking a comprehensive research programme to assess the extent of ASPs starting with hospitals within a key province in Pakistan. The findings can be used to develop pertinent programmes to address concerns not only in Punjab but across Pakistan where they exist.

## 2 Methodology

### 2.1 Study design and instrument

In this quantitative observational study, a survey was conducted to evaluate the number of AMS practices and general ASP activities in Pakistan as well as their perceived impact. An instrument of measure was developed based on health care facility characteristics and ASPs following an extensive literature review [11, 12, 14, 19]. The types of ASPs were broadly grouped into: administration related activities and antimicrobial use and prescribing-related activities. The participants subsequently related the perceived effectiveness of the different activities using a 5-point measure ranging from unsuccessful, somewhat successful, moderately successful, very and successful and extremely successful. Face and content validity of the questionnaire was performed by experts in the field of quantitative research. Faced validity was undertaken to uncover obvious problems and check the relevance of the questionnaire as it appeared to respondents. In order to cover all relevant issues, content validity was performed through an extensive literature review. Cronbach's alpha was used to determine the average correlation of items or internal consistency in the survey instrument to gauge its reliability. The value of Cronbach's alpha was 0.791, which is considered acceptable in social science research. Before full scale study, a pilot study was undertaken and pertinent changes were made to the survey instrument by removing suggested flaws accordingly. The study was approved by the University College of Pharmacy, University of the Punjab, Ethics Committee on Human Research (HEC/1000/PUCP/1925SAMS).

### 2.2 Inclusion criteria and survey instrument

Punjab, the largest and most populous province, was chosen for this initial study as a representative area of Pakistan. The survey instrument was piloted and validated by practicing hospital pharmacists, physicians and pharmacists/physicians in research and academia before full implementation. This survey included a variety of facility types representing the different facilities in Pakistan. All private, charity and public sector hospitals including primary, secondary, tertiary, and specialized care hospitals, were contacted. Contact details were obtained from the Director General Health Services office, Department of Health, Government of the Punjab, Pakistan. This survey was initially sent out to acute care facilities (ACFs) with >25 beds. Health care settings with <25 beds were not included as they are typically located in rural areas, have inadequate health care services, and are generally linked with a larger health care settings to encourage patient transfer when required.

### 2.3 Data collection and analysis

The survey questionnaire was circulated by mail or through drop off survey to medical superintendents or directors/heads of pharmacy departments from February to May 2018. An introductory e-mail or cover letter was also sent to highlight the nature and importance of this survey. The participants were ensured that all collected data would be anonymized by using codes in order to ensure confidentiality. Only one representative from each hospital was invited to complete the survey. The participants were also requested, where appropriate, to disseminate this survey link through their contacts or organisations. Either the medical superintendents or directors of pharmacy departments personally, or their representative, completed the survey. After 2–3 weeks, the first reminder was forwarded with the second and final reminder sent after a further three weeks. Data were analyzed using the latest versions of Microsoft Excel and SPSS (version 22 IBM, California, USA). Descriptive statistics (frequency and percentages) was applied on categorical variables.

## 3 Results

The response rate was 62.1%, i.e. 168 completed survey forms were obtained out of a total of 267 sent out. The highest response rate was seen among public sector hospitals (109, 79.5%) followed by charity (24, 61.5%) and private sector hospitals (35, 44.9%). From the total number of surveys received, 31 entries were subsequently excluded because the respondents did not record the information properly. This left a total of 137 hospitals who provided the complete information contained in the questionnaire: 11 primary, 65 secondary and 61 tertiary hospitals. Pharmacists accounted for 55.5% (76/137) of the survey returns, physicians for 22.6% (31/137) and medical superintendents for 21.9% (30/137). The details of personnel taking part and hospital information are given in Table 1.

**Table 1:** Personal information of respondents.

Characteristics	Number	Percentage
**PERSONAL INFORMATION**		
**Gender**		
Male	88	64.2
Female	49	35.8
**Level of qualification/Terminal Degree**		
Bachelor	75	54.7
Master or Fellowship	62	45.3
**Designation**		
Pharmacist	76	55.5
Physicians	31	22.6
Medical Superintendent	30	21.9
**HOSPITAL INFORMATION**		
**Hospital Type**		
Primary	11	8.0
Secondary	65	47.4
Tertiary	61	44.5
**Hospital Ownership**		
Charity	17	12.4
Private	28	20.4
Public	92	67.2
**Area**		
Rural	37	27.0
Urban	100	73.0

The types of ASPs activities are shown in Tables 2 and 3. The use of Drug and Therapeutics Committees (DTCs) or equivalent committees to approve antimicrobial use (65.5%) was the most common administration-related activity. This was followed by regular auditing by doctors regarding their antimicrobial prescribing and use (54.1%) and the use of a restricted formulary for antimicrobials within the hospital (50.4%). The overall perceived rate of activities to become usuccessful was very low. However, most of the documented activities were only somewhat or moderately successful. Only 19.7% of hospitals participated in the national antimicrobial utilization surveillance program. Nearly 25% of hospitals had established a mechanism for conflict resolution in an event of a disagreement with respect to the prescribing of antimicrobials between practitioners and computerized clinical decision support systems when integrated into the health records at the time of prescribing. Within antimicrobial use and prescribing-related activities, most of the respondents commented that they use clinical guidelines to guide their prescribing (68.7%). The provision of infectious diseases consultation services by infectious diseases clinicians was witnessed in 66.4% of hospitals. Clinical pharmacy service was provided in 65.7% of healthcare facilities. Streamlining or de-escalation (treatment is re-directed after culture results have been obtained) of antimicrobial therapy and timely conversion of IV to oral antimicrobials were also very common activities. These activities were perceived to be somewhat very successful by respondents. Electronic antimicrobial prescribing approval systems were used in only 15.3% of hospitals surveyed, and only 16.1% of hospitals were using a sticker to notify prescribers regarding the need to obtain approval for the antimicrobial prescribed.

**Table 2:** Administration-related antimicrobial stewardship activities.

Administration-related activities	No. of Respondents - %s in brackets			Perceived success of the activity (only those who answered “Yes” to having the activity at their institution – with %s vs. total in brackets)				
	Don't know	No	Yes	Unsuccessful	Somewhat successful	Moderately successful	Very successful	Extremely successful
Drug and Therapeutic Committee or equivalent Committee approving antimicrobials listing on the formulary and its associated use	13 (9.5)	35 (25.5)	89 (65.5)	3 (2.2)	35 (25.5)	21 (15.3)	22 (16.1)	8 (5.8)
Restricted formulary for antimicrobial prescribing	17 (12.4)	51 (37.2)	69 (50.4)	4 (2.9)	17 (12.4)	25 (18.2)	14 (10.2)	9 (6.6)
Locally formulated antimicrobial policy	12 (8.8)	64 (46.5)	61 (44.5)	6 (4.4)	23 (16.8)	11 (8.0)	15 (10.9)	6 (4.4)
Regular audit by administrators on the prescribing and use of antimicrobials	9 (6.6)	68 (49.6)	60 (43.8)	7 (5.1)	16 (11.7)	10 (7.3)	19 (13.9)	8 (5.8)
Regular audit by doctors on antimicrobial prescribing and use	15 (10.9)	48 (35.0)	74 (54.1)	7 (5.1)	16 (11.7)	19 (13.9)	20 (14.6)	12 (8.8)
Regular audit by pharmacists on antimicrobial prescribing and use	8 (5.8)	62 (45.3)	67 (48.9)	1 (0.7)	19 (13.9)	20 (14.6)	12 (8.8)	15 (10.9)
Regular audit by nurses on antimicrobial prescribing and use	14 (10.2)	61 (44.5)	62 (45.3)	5 (3.6)	22 (16.6)	12 (8.8)	17 (12.4)	6 (4.4)
Established mechanism for conflict resolution in event of disagreement with respect to use of antimicrobials between practitioners	17 (12.4)	87 (63.5)	33 (24.1)	–	12 (8.8)	8 (5.8)	9 (6.6)	4 (2.9)
Participation in the National Antimicrobial utilization Surveillance Program	14 (10.2)	96 (70.1)	27 (19.7)	2 (1.5)	7 (5.1)	6 (4.4)	12 (8.8)	–
Multidisciplinary antimicrobial stewardship team or equivalent to coordinate activities in hospital	6 (4.4)	89 (65.0)	42 (30.7)	5 (3.6)	10 (7.3)	13 (9.5)	14 (10.2)	–
Computerized Clinical Decision Support Systems Integrated into the Health Record at the Time of Prescribing	7 (5.1)	96 (70.1)	34 (24.8)	–	3 (2.2)	12 (8.8)	4 (2.9)	15 (10.9)
Routine access to an infectious disease specialist (even in another hospital if needed)	14 (10.2)	62 (45.3)	61 (44.5)	5 (3.6)	11 (8.0)	13 (9.5)	25 (18.2)	7 (5.1)
Routine availability of reagents and discs to perform sensitivity analyses of specimens	11 (8.0)	62 (45.3)	64 (46.5)	1 (0.7)	21 (15.3)	21 (15.3)	14 (10.2)	7 (5.1)
Work with the Microbiology Laboratory to Develop Stratified antibiograms	14 (10.2)	82 (59.9)	41 (29.9)	–	10 (7.3)	12 (8.8)	11 (8.0)	8 (5.8)
Antibiograms used to develop guidance for the empiric use of antibiotics within the hospital	7 (5.1)	93 (67.9)	37 (27.0)	–	11 (8.0)	11 (8.0)	5 (3.6)	10 (7.3)
Advocate for Rapid Diagnostic Testing for bacteria and viruses to Optimize Antibiotic Therapy	9 (6.6)	65 (47.4)	63 (46.0)	4 (2.9)	21 (15.3)	16 (11.7)	14 (10.2)	8 (5.8)
Develop Facility-Specific Clinical Guidelines for Management of Fever and Neutropenia (F&N)	11 (8.0)	78 (56.9)	48 (35.0)	4 (2.9)	15 (10.9)	13 (9.5)	4 (2.9)	12 (8.8)

**Table 3:** Antimicrobial use and prescribing-related antimicrobial stewardship activities.

Antimicrobial use and prescribing-related activities	No. of Respondents - %s in brackets			Perceived success of the activity (only those who answered “Yes” to having the activity at their institution - with %s vs. total in brackets)				
	Don't know	No	Yes	Unsuccessful	Somewhat successful	Moderately successful	Very successful	Extremely successful
Provision of clinical pharmacy services	3 (2.2)	44 (32.1)	90 (65.7)	11 (8.0)	22 (16.1)	27 (19.7)	22 (16.1)	8 (5.8)
Provision of consult service by Infectious Diseases Clinicians	12 (8.8)	34 (24.8)	91 (66.4)	17 (12.4)	24 (17.5)	23 (16.8)	21 (15.3)	6 (4.4)
Streamlining or de-escalation of therapy (i.e. treatment is re-directed after culture results have been obtained)	3 (2.2)	51 (37.2)	83 (60.1)	2 (1.5)	23 (16.8)	20 (14.6)	24 (17.5)	14 (10.2)
Use of clinical guidelines to guide antimicrobial prescribing	13 (9.5)	30 (21.9)	94 (68.7)	2 (1.5)	33 (24.1)	35 (25.5)	20 (14.6)	14 (10.2)
Program for timely conversion of IV to oral antimicrobials	5 (3.6)	50 (36.5)	82 (59.5)	–	32 (23.4)	19 (13.9)	20 (14.6)	11 (8.0)
Use of phone-based approval system for antimicrobial prescribing	3 (2.2)	93 (67.9)	41 (30.0)	2 (1.5)	15 (10.9)	13 (9.5)	5 (3.6)	6 (4.4)
Regular multidisciplinary antimicrobial stewardship ward round to some wards or for certain patient groups	8 (5.8)	89 (65.0)	40 (29.2)	2 (1.5)	15 (10.9)	5 (3.6)	12 (8.8)	6 (4.4)
Use of a sticker to notify prescribers regarding the need to obtain approval for antimicrobial prescribed	1 (0.7)	114 (83.2)	22 (16.1)	–	12 (8.8)	4 (2.9)	6 (4.4)	–
Use of automatic ‘stop orders’ for antimicrobials prescribed	1 (0.7)	98 (71.5)	38 (27.7)	2 (1.5)	8 (5.8)	17 (12.4)	3 (2.2)	8 (5.8)
Use of electronic antimicrobial prescribing approval systems	2 (1.5)	114 (83.2)	21 (15.3)	2 (1.5)	4 (2.9)	2 (1.5)	1 (0.7)	12 (8.8)
Rotation of selected antimicrobial drugs within a specific timeframe	12 (8.8)	64 (46.7)	61 (44.5)	3 (2.2)	15 (10.9)	19 (13.9)	17 (12.4)	7 (5.1)
Implement Interventions to Reduce the Risk of Antibiotics Associated Clostridium Difficile Infection	18 (13.1)	66 (48.2)	53 (38.7)	3 (2.2)	17 (12.4)	8 (5.8)	16 (11.7)	9 (6.6)
Implement Strategies That Promote Cycling or Mixing in Antibiotic Selection to Reduce Antibiotic Resistance	7 (5.1)	55 (40.1)	75 (54.7)	6 (4.4)	23 (16.8)	20 (14.6)	19 (13.9)	7 (5.1)
Dedicated Pharmacokinetic (PK) Adjustment/TDM Program Lead to Improved Clinical Outcomes and Reduced Costs	8 (5.8)	94 (68.6)	35 (25.5)	–	9 (6.6)	7 (5.1)	11 (8.0)	8 (5.8)
Advocate C-Reactive Protein (CRP), Procalcitonin (PCT) Testing	19 (13.9)	74 (54.0)	44 (32.1)	2 (1.5)	7 (5.1)	12 (8.8)	11 (8.0)	12 (8.8)

## 4 Discussion

This snapshot has highlighted important results on the exsietnce of ASPs among private and public sector hospitals in the Punjab region of Pakistan. Disappointingly, only a limited number hospitals have successfully instigated ASP which is a concern for hospitalized patients within a country [3]. This has also been seen among Pakistani hospitals, which had not yet fully started ASP initiatives [2], reflecting LMICs generally, which have lower levels of ASPs than developed countries enhanced by the absence of necessary infrastructures and political commitment [9, 20]. However, it is encouraging that ASPs are being developed, and appear successful in LMIC to tackle the challenge of AMR [10, 21, 22]. Whereas, ASPs are well established in developed countries which possibly reveals strong historical commitment and leadership on ASP from their organizations [11, 12].

Regardless of the presence of large well-established public and private hospitals across Pakistan, the present study showed that only a few ASPs exist in most of the hospitals. This may reflect limited knowledge of ASPs generally among physicians in hospitals in Pakistan including those working in tertiary hospitals [23]. The present study also showed that four-fifths of facilities failed to participate in the national antimicrobial utilization surveillance program, indicating that the data generated in that program may not be representative of the nation as a whole. Moreover, this survey also highlighted that the success rate of antimicrobial stewardship activities is very low, which needs to be addressed going forward.

The study also showed discrepancies in standard approaches to ASPs that need to be addressd in Pakistani hospitals in the future. The published literature documents that pharmacist services are frequently used to rationalize dosing, implement IV-to-oral switching or support post-prescription reviews to improve future antibiotic use in hospitals, while infectious disease specialists are essential for diagnostic input and ward rounds [24]. However, the possible valuable role of nurses in ASP is typically underestimated [25].

In this present study, it was also noted that in most hospitals, there were limited diagnostic facilities and that the availability of reagents and development of antibiogram was uncommon. This is a concern and suggests prescribing of antimicrobials was mainly empiric. In addition, as the role of the medical microbiologist and the microbiology laboratory are central to any ASP, mainly through the development of antibiograms [26], with increasing use of diagnostic interventions shown to decrease the burden of unnecessary antimicrobial use [27]. Antimicrobial stewardship ward rounds have also been regularly used to reduce irrational antimicrobial prescribing and pharmacy cost as well as reduce infection rates and AMR [28]. Our study results suggest that most of the hospitals taking part did not have institutional prescribing guidelines. However, encouragingly, the approval of restricted formulary for antimicrobials through DTCs or equivalent committees within the hospital and regular auditing of antimicrobial prescribing and use was common among the participating hospitals. This means the majority of hospitals have DTCs to be able to influence prescribing as we see in other countries [29, 30]. We are also aware that regular audituing and feedback have beenused to successfully as a cost effective way to restrict the use of broad-spectrum antibiotics [31]. Antibiotic mixing or cycling strategies have also occasionally been reported, probably reflecting the practical problems in undertaking this, including scarcity of evidence to support mixing and considerable use of resources [32, 33]. . In addition, the effective use of multidisciplinary models for implementing ASPs helps achievethe required outcomes. Competency and outcomes based lectures, workshops, lunchtime presentations, face–to–face interventions, e-learning and other possible educational framework for doctors, nurses and pharmacists are useful in planning the implementation of ASPs [11]. However, there is a need to focus on a minimum set of achievable targets, which is especially relevant to Pakistan. In addition, address the major issue of diagnostic facilities and professional advice through microbiologists and infectious disease specialists.

We are aware that there are several limitations of our survey. First, the recruitment of respondents was through professional associations and contact sources of the authors, contributing to potential recruitment bias. Second, there was a difference between the regions in terms of reporting results. The number of returns from central Punjab was high and well-representative as compared to north and south Punjab. Third, as most of the surveys were addressed to Chief Pharmacist or their nominees, the responses received could have been biased towards the pharmacists' perspectives. Fourth, there could be self-bias, particularly in case private sector hospitals, either not willing or incorrect information. We also did not collect the complete data of hospital demography including the number of beds, number of admissions/day, number of intensive care units, laboratory testing capacity. Last, there was only a moderate response rate and we only induded hospitals in Punjab limiting the generalizing of the results to all hospitals in Pakistan. Never-the-less, we believe that in view of our methodology the findings are rov = bust and provide clear direction to improve the use of antimicrobials in hospitals in Pakistan in the future.

## 5 Conclusion

The study inferred that there are inadequate ASPs among the hospitals of Pakistan. A multidisciplinary approach, clinical leadership and the availability of enthusiastic and motivated individuals are essential elements for the success of ASPs. Adequate clinical pharmacy and infectious disease resources should be made available by the government to support ASPs in the future as the government moves forward with its strategy to reduce AMR rates in Pakistan. We hope that this work will inform local and international policy-makers about current stewardship activities and challenges with a view to foster broader international collaboration as recommended by the WHO.

## Declarations

### Author contribution statement

Zikria Saleem: Performed the experiments; Wrote the paper.

Mohamed Azmi Hassali: Conceived and designed the experiments.

Furqan Khurshid Hashmi: Performed the experiments.

Brian Godman: Analyzed and interpreted the data; Wrote the paper.

Zakkiudin Ahmed: Analyzed and interpreted the data.

### Funding statement

This research did not receive any specific grant from funding agencies in the public, commercial, or not-for-profit sectors.

### Competing interest statement

The authors declare no conflict of interest.

### Additional information

No additional information is available for this paper.
